# The Use of the Mallet

**Published:** 1863-05

**Authors:** W. H. Atkinson


### THE USE OF THE MALLET.

BY DR. W. H. ATKINSON.

(Read before the New York Society of Dental Surgeons, November 20, 1861.)

Having been politely requested to give an outline of the method pursued by me in filling teeth with the mallet, I come before you to-night, gentlemen, in the spirit of a true fraternity, and of earnest enquiry, hoping to impress each one who hears me with that freedom from restraint that always tends to kill interested and partial statements and propositions. If any man living has need to be free from half-truth and fractional or ambiguous propositions, it is he who is engaged in demonstrative teaching; and that we may at once lay aside all the dead weights which weak minded and selfish men have thrown around professional subjects, let us consider the subjects presented to our view in a searching and kindly spirit of investigation.

It has heretofore been supposed that a certain reserve, and what has been called dignity, should attach to every one occupying a chair or professorship in any faculty, for the honor of the faculty and profession, going upon the assumption that "familiarity breeds contempt," which I utterly repudiate as principle or as fact. If it were true, I would myself be most unfit to teach the least of all sciences; for I frankly confess to you at the outstart that I know but very little of the vast field open in the direction of demonstrative teaching of scientific truth and philosophy. But that we do not become unnecessarily discouraged, I will state that it is not that any know so much, but that the masses of investigators know so very little, that makes us sometimes think the hiatus so great between teacher and learner. My first proposition for the encouragement of my own heart, and the hearts of all desirous to learn, is "Whatsoever sane human minds have done sane human minds may again do." But to accomplish the same amount of work alone, and without the assistance of a competent teacher or pioneer, will cost us much more time, effort and other expenditures. Hence, it is the highest wisdom of both teacher and learner to come together in such real earnest as to create an atmosphere of such enquiry and ready response as to invite us out of our self-hood by the intensified attention to whatever we may have in hand for construction or dissection to such degree that our interests may become a unit. And by this first step being fully taken we shall soon find ourselves in the true line of discovery, or apprehension of truths yet not taught or even apprehended. Were this not so, no advance of pupil beyond the dead letter of teacher's text could be possible. I say it, gentlemen, considerately, as truly that to this I owe more of whatever advance I have made than to all the authorities and so-called leaders, in the various branches at which it has been my fortune to glance. The dead text in a book you con not put to the test of cross-examination, neither can you approach the same-time professors and teachers of science. But, thank God, the time has come when high-sounding titles and long tails affixed to men's names can not screen them from the sharp scrutiny of fresh and vigorous minds, if they assume to teach demonstrable branches.

The Dental branch of science is, par excellence, the demonstrative department in which shallow pretence can in no wise long hide its deformity. In Medicine, Law, and Divinity teaching has become so completely bedeviled and befogged that it requires as much ability and learning to detect as to perpetuate the errors denominated regular normal practice in any and all of them. By the especial favor of heaven has this department been ushered into existence in its present necessities and nakedness of form, for the fulfilment of law heretofore substituting and holding relation to this department, just as Judaism did to Christianity. And, thanks be to God, the so-called publicans and sinners of the profession, like the gentiles and conquerors of old, accepted the truth of the dental department readily, because they had no false record, or false dignity, or self-righteousness to interpose between their eyes and the truth presented, and they were at once soundly converted, while the well educated Anatomists, Physiologists and Pathologists, like the Jews, having had the law in each of these branches before them from the earliest times, had unwittingly passed lightly over the teeth and their important and paramount relations, set about to persecute, ignore and crucify this reproachful young reformer, claiming such right to fulfil, and thereby set aside the former law, by instituting his new advent, that assumed to be the focalizing point of all the basal principles, whose reign held the government in all that had preceded this new Lord and Master of Science. To be sure, the first apostles were of the fishermen artizans and uneducated classes at the first, out of whom many devils of ignorance, weakness and malice had to be cast before they were even tolerably decent representatives of the high artistic skill and great utilitarian practices of preventing physical, mental and moral aberrations which lay the foundation for, and create the necessity to have the former three soon to be absolete professions of Law, Medicine and Divinity, which, like the Jewish dispensation, giving way to the great teacher fulfilling his mission, which included in one synthetic practice of godliness, the tedious voluminous, expensive and divine representatives of rites and ordinances, must be annihilated in the wholeness of a true professional life, which consists in teaching the race how to avoid all aberrations from the normal systemic action by strict and ready spontaneous obedience to the laws of the economy. But as there still are Jews and Pagans on earth, amid the full blaze of Gospel day, so it will be a long road in the ordinary sense, that poor humanity will have to travel before all will have the grace, good sense and humility to kindly take to the truth, and live it out in strict obedience to their first and highest convictions of the truth and purity of an unselfish life.

Since from this view of the case, it will be necessary for some of us to practice a little longer, I will endeavor to make out a plain path for our feet.

1st. When it is necessary to understand the laws of the upbuilding of teeth, and thus become acquainted with the anatomy or foundation of our subject; and as this is of itself an herculean labor, you of course will not require me to enter upon it in this short address.

2d. After normal, complete structures are understood, the laws of disintegration will next claim attention.

3d. Then the best means of prevention will be our highest function; and our next highest will be the one great necessity of the masses who call upon the practitioner of a true, dental science, viz: Arrestation of the disintegrative process, usually called decay caries, "rotten teeth."

This last endeavor has occupied my feelings, ideas, thoughts, opinions, beliefs and knowledge more than any other speciality of science. It involves some knowledge of chemistry and much mechanical skill with the equipoisity of the distribution of force, &c., &c. I have discovered that the force produced must be directly applied, and exactly expended or lateralized in the process of packing the filling to prevent the impetus from being felt by the patient, so that if we could justly estimate and apply the force requisite to consolidate the filling to the degree of resistance of the tooth-substance, we could fill the tooth without the patient being aware of it from the sensation produced. But as this is a nicety of admeasurement of force that no dentist that I have ever heard of has yet attained, I would advocate the nearset approach to this accuracy known to me. This is attained by a well constructed mallet, in the hand of intelligence. And as words alone can hardly be deemed adequate to convey a just apprehension of the truly wonderful results thus attained, I must defer the demonstration until I can address two senses, viz: both the eye and ear.

I now, as my custom is, will pause for any direct question that any may wish to propose.

